# Specific induction and long-term maintenance of high purity ventricular cardiomyocytes from human induced pluripotent stem cells

**DOI:** 10.1371/journal.pone.0241287

**Published:** 2020-11-02

**Authors:** Hiroyuki Fukushima, Miki Yoshioka, Masahide Kawatou, Víctor López-Dávila, Masafumi Takeda, Yasunari Kanda, Yuko Sekino, Yoshinori Yoshida, Jun K. Yamashita

**Affiliations:** 1 Department of Cell Growth and Differentiation, Laboratory of Stem Cell Differentiation, Center for iPS Cell Research and Application (CiRA), Kyoto University, Kyoto, Japan; 2 Department of Cardiovascular Surgery, Kyoto University Graduate School of Medicine, Kyoto, Japan; 3 Institute for Advancement of Clinical and Translational Science (iACT), Kyoto University Hospital, Kyoto, Japan; 4 Division of Pharmacology, National Institute of Health Sciences, Kawasaki, Kanagawa, Japan; 5 Department of Cell Growth and Differentiation, Center for iPS Cell Research and Application (CiRA), Kyoto University, Kyoto, Japan; University of Tampere, FINLAND

## Abstract

Currently, cardiomyocyte (CM) differentiation methods require a purification step after CM induction to ensure the high purity of the cell population. Here we show an improved human CM differentiation protocol with which high-purity ventricular-type CMs can be obtained and maintained without any CM purification process. We induced and collected a mesodermal cell population (platelet-derived growth factor receptor-α (PDGFRα)-positive cells) that can respond to CM differentiation cues, and then stimulated CM differentiation by means of Wnt inhibition. This method reproducibly generated CMs with purities above 95% in several human pluripotent stem cell lines. Furthermore, these CM populations were maintained in culture at such high purity without any further CM purification step for over 200 days. The majority of these CMs (>95%) exhibited a ventricular-like phenotype with a tendency to structural and electrophysiological maturation, including T-tubule-like structure formation and the ability to respond to QT prolongation drugs. This is a simple and valuable method to stably generate CM populations suitable for cardiac toxicology testing, disease modeling and regenerative medicine.

## Introduction

The generation of cardiomyocytes (CMs) from human pluripotent stem cells (hPSCs), including human embryonic stem cells (hESCs) and induced pluripotent stem cells (hiPSCs), has been increasingly explored for various applications, such as cardiotoxicity screening, drug discovery, disease modeling, as well as regenerative medicine [1–3]. The generation of pure and stable human CM populations is a fundamental requirement to meet the wide CM demand. Similarly, developing methods for the long-term maintenance of high-purity, structurally and electrophysiologically mature ventricular cardiomyocytes is essential to improve cardiac toxicological and pharmacological studies.

Although recent advances in CM differentiation protocols robustly and efficiently yield CMs, most available protocols still need some kind of purification step at the final stage of CM differentiation, such as the Percoll density gradient procedure [4, 5], genetic manipulation using cardiac-specific promoters [6–8], cell sorting using mitochondrial dyes [9], microRNA-regulated fluorescence [10], or antibodies directed against cardiac cell surface markers such as signal-regulatory protein alpha (SIRPA) [11] and vascular cell adhesion molecule 1 (VCAM1) [11, 12], or metabolic selection using glucose-depleted culture medium containing lactate [13]. Even after CM purification, the resulting purity can be reduced during long-term cultures that span several months [14]. This is likely because CM purification after terminal differentiation does not fully guarantee the abscence of other cell types. In addition, the purification process might cause some mechanical and physiological stress to the cells that may reduce the quality and viability of the purified CM population. Removal of non-suitable cells at the earlier stages of the CM differentiation process might be effective for the long-term maintenance of high-purity CM populations.

We have previously reported methods both for efficient CM differentiation and for the simultaneous differentiation of several cardiovascular cell types [12, 15] based on a high-density hESC monolayer culture [5]. After mesoderm was induced by treating the cells with Activin A (ActA) for 24 hours, followed by bone morphogenetic protein 4 (BMP4) and basic fibroblast growth factor (bFGF) for 4 days, Wnt signal inhibitors such as Dickkopf-related protein 1 (Dkk1) were used to efficiently induce CM commitment [12, 16, 17]. Vascular endothelial growth factor (VEGF) was added instead of Wnt inhibitors for the simultaneous induction of endothelial cells (EC), pericytes and CMs [15]. In addition to these methods for CM differentiation, we previously developed a robust and efficient differentiation method for the induction of ECs from hiPSCs with extremely high efficiency (>99%). This method involves the purification of VEGF receptor-2 (VEGFR2)-positive (VEGFR2^+^) cells, which are responder cells to EC commitment signals, to eliminate non-responder cells at the early mesoderm stage (stimulation-elimination [SE] method) [18].

The highly specific induction of ECs by the SE method prompted us to examine and apply the SE approach to CM differentiation. In this study, we selected platelet-derived growth factor receptor-α (PDGFRα) as a marker for cells susceptible to CM differentiation cues. PDGFRα is highly expressed in paraxial mesoderm [19] and involved in cardiovascular tissue development in mouse embryo [20]. PDGFRα has often been reported as a marker for cardiac mesoderm and cardiovascular progenitors that give rise to CMs during both mouse and human PSC differentiation [21–26]. We recently reported that PDGFRα^+^ cells derived from hiPSCs possess high CM differentiation potential [16] Thus, we induced and purified PDGFRα^+^ mesoderm cells at differentiation day 5 (d5) and re-cultured them in medium containing Wnt inhibitors (XAV939 and IWP4) to prompt specific commitment to the CM lineage. With this approach, we succeeded in establishing a method for high-purity CM differentiation (>95%) without any CM purification steps, as well as ensuring the long-term maintenance (more than 200 days) of such high-purity CM population. These CMs showed a predominantly ventricular phenotype and a tendency towards structural and electrophysiological maturation during long-term maintenance. We further demonstrated the utility of these long term-cultured CMs for pharmacological studies.

## Materials and methods

### Human iPSC lines

The 836B3 hiPSC line, established from an adult fibroblast cell line transfected with 6 factors (Oct3/4, Sox2, Klf4, L-Myc, LIN28, and Glis1) in episomal plasmid vectors [27], was used as a representative human iPSC line in all experiments unless stated otherwise. The 253G1 hiPSC line, established from skin fibroblasts following the retroviral transfection of 3-factors (Oct3/4, Sox2 and Klf4) [28], and the 1201C1 hiPSC line, established from an adult peripheral blood cell line transfected with 6 factors (Oct3/4, Sox2, Klf4, L-Myc, LIN28, and shRNA for TP53) in episomal plasmid vectors [29], were utilized to study the induction of CMs and the maintenance of CM purity. All hiPSC lines were originally established at and provided by the Center for iPS Cell Research and Application (CiRA), Kyoto University, Kyoto, Japan.

### Human iPSC maintenance

The hiPSC maintenance culture method utilized here has been previously reported [5, 12, 15]. In brief, hiPSCs were maintained on a matrigel (#354230, Growth factor reduced, 1:60 dilution; Corning), Eugene, OR)-coated dish in mouse embryonic fibroblast conditioned medium (MEF-CM) supplemented with 4 ng/mL human basic fibroblast growth factor (bFGF; #068–04544, WAKO, Osaka, Japan). Human iPSCs were passaged as small clusters approximately every 5 days using CTK solution (0.1% collagenase IV, 0.25% Trypsin, 20% knockout serum replacement and 1mM CaCl_2_ in phosphate buffered saline).

### CM differentiation

**Fig 1:**
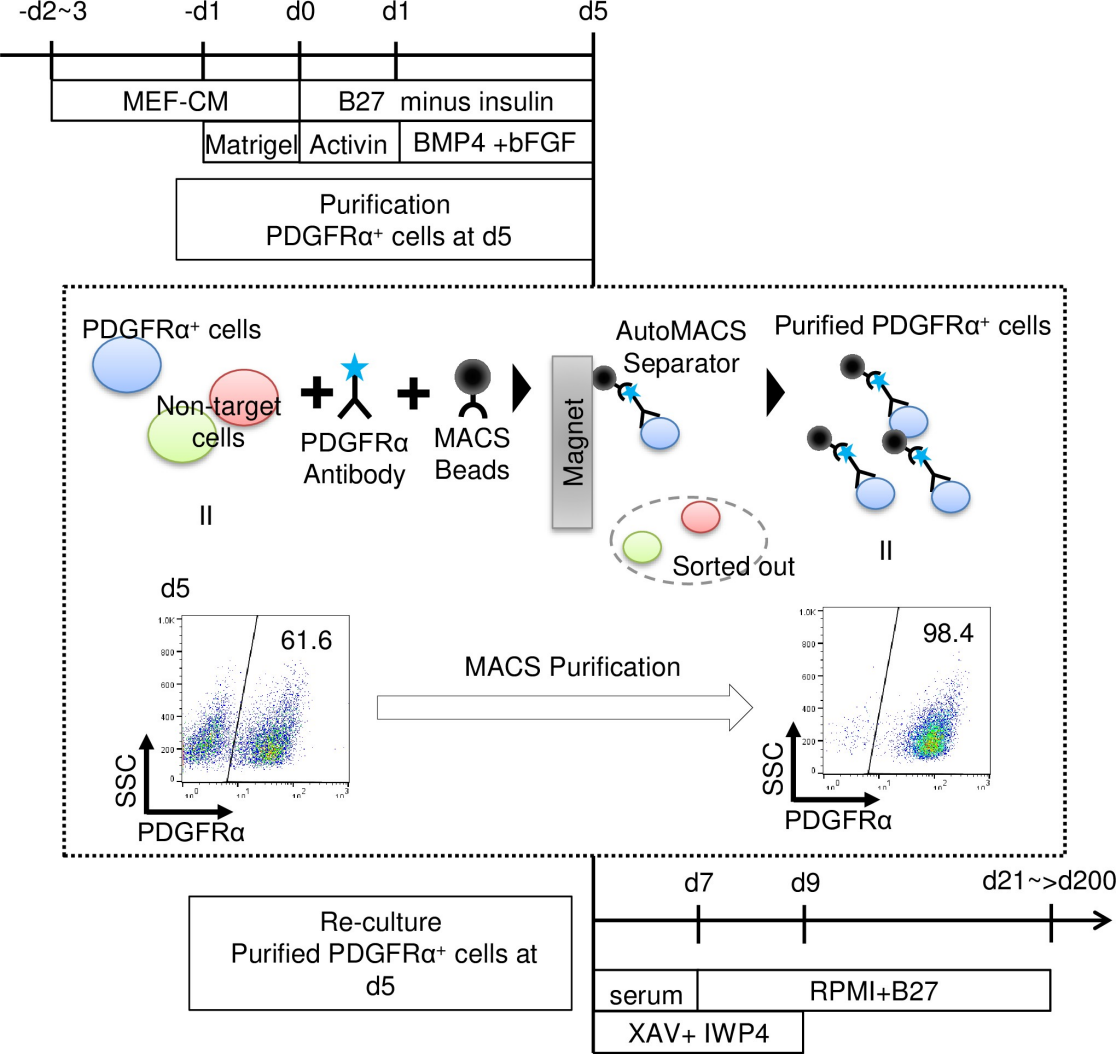
Scheme of optimized CM differentiation protocol.

The CM differentiation protocol utilized here is a minimally modified version of a previously reported protocol [5, 12, 15] and is shown in Fig 1. hiPSCs were treated with Versene (#15040066, 0.48 mM EDTA solution; Thermo Fisher Scientific) and incubated for 3–5 min at 37°C. Then Versene was aspirated and MEF-CM was added. Cells were detached using a cell scraper (#MS-93100, SUMILON), collected and counted, before being seeded onto the matrigel-coated dish described above at a high density of 1.0–1.5×10^5^ cells/cm^2^ in MEF-CM (0.52 mL/cm^2^) supplemented with 4 ng/mL bFGF, and cultured for 2–3 days before induction. On the day before induction (-d1), after confirming cells were fully confluent, medium was changed to MEF-CM supplemented with 4 ng/mL bFGF and matrigel (0.52 mL/cm^2^) (#354230, Growth factor reduced; 1:60 dilution; Corning). To induce cardiac differentiation, we switched from MEF-CM to RPMI1640 (#21870092, Thermo Fisher Scientific) medium with B27 supplement minus the insulin (insulin-; A1895601, Thermo Fisher Scientific) (RPMI1640, 2 mM L-glutamine, 1×B27 supplement without insulin) containing 100 ng/mL Activin A (ActA; #338-AC, R&D systems, Minneapolis, MN) on day 0 (d0), and incubated cells for 24 hours. Then medium was changed to RPMI1640, 2 mM L-glutamine, with 1×B27 supplement without insulin containing 10 ng/mL human bone morphogenetic protein 4 (BMP4; #314-BP, R&D) and 10 ng/mL bFGF, and cells were incubated for 4 days without changing medium.

On day5, PDGFRα^+^ cells were purified using magnetic-activated cell sorting (MACS)(see Purification of PDGFRα^+^ cells with MACS), plated onto gelatin or matrigel-coated plates at a density of 3.12–3.65 × 10^5^ cells/cm^2^ in RPMI1640 supplemented with 10% fetal bovine serum (FBS, Lot.8D0264, Cell Culture Bioscience, NICHIREI BIOSCIENCES Inc., Tokyo, Japan) and 2 mM L-glutamine containing Wnt inhibitors, XAV939 (10 μM) (#575545, Millipore, Billerica, MA) and/or IWP4 (5 μM) (#040036, Miltenyi Biotec, Bergisch Gladbach, Germany), and a ROCK inhibitor (Y-27632, WAKO, 10–20 μM) (#251–00514, WAKO) and cultured for 2 days. On day7, culture medium was changed to RPMI1640 with regular B27 supplement (#17504044, Thermo Fisher Scientific) with a minimal dose of Wnt inhibitors, XAV939 (0.25 μM) and IWP4 (0.125 μM), and cells incubated for 2 days. On day 9, culture medium was changed to RPMI1640 with B27 supplement alone and refreshed every 2–3 days.

### Long-term culture of CMs

On d21, CMs were detached using 0.25% Trypsin-EDTA for 10-15min at 37°C, and reseeded on a culture dish at 2.0 × 10^5^ cells/cm^2^ with alpha minimum essential medium (αMEM; #11900–024, Thermo Fisher Scientific) supplemented with 2.2 g/L NaHCO_3_, 10% FBS and 5 × 10^−5^ M 2-mercaptoethanol and containing Y-27632 (10 μM) and cultured for 2 days. Culture dishes were prepared by pre-coating them with Poly-L-lysine (5 μg/mL, Sigma P1399) at room temperature for 20 min. After thoroughly rinsing with PBS(-), dishes were coated with 0.5% gelatin (Sigma) or matrigel (Growth factor reduced, 1:60 dilution; Corning) overnight at 4°C. After two days of cell incubation, culture medium was replaced by RPMI1640 with B27 supplement and refreshed every 2–3 days. Cells were passaged every 20 days.

To assess CM proliferation, CMs were detached and collected after treatment with 0.25% trypsin/EDTA (#25200, Thermo Fisher Scientific), and then viable CMs were counted using a hemocytometer after 0.4% trypan blue staining (#15250061, Thermo Fisher Scientific). CMs on d21 were seeded at a 2.0 × 10^5^ cells/cm^2^ density (1.9 × 10^6^ cells per well of a 6-well plate), and the accumulated cell number was calculated from counting viable CM numbers at each cell passage.

### Magnetic Activated Cell Sorting (MACS)

On day5, cells were dissociated by incubation with Accumax (#AM105-500, Innovative Cell Technologies, San Diego, USA) for 20-30min at 37°C. The dissociated cells were then centrifuged and washed twice with HBSS containing 0.1% BSA and 0.35g/L NaHCO_3_ (HBSS/BSA; Thermo Fisher Scientific), and suspended in 70 μL of HBSS/BSA for every 1 × 10^7^ cells. Cells were stained with an anti-PDGFRα antibody conjugated to allophycocyanin (APC) (#FAB1264A, mouse monoclonal, clone PRa292, R&D) (1 μL per 1 × 10^6^ cells) for 20–30 min at room temperature. The labeled cells were then washed twice with HBSS/BSA, and then suspended in 70 μL of HBSS/BSA for every 1 × 10^7^ cells. Then, 20 μL of anti-APC microbeads (#130-090-855, Miltenyi Biotec) for every 1 × 10^7^ cells were added to the mix. The suspended cells were incubated for 20–30 min at room temperature and washed twice with HBSS/BSA. PDGFRα^+^ cells were, thus, magnetically labeled by an APC-conjugated anti-PDGFRα antibody and anti-APC microbeads. Lastly, the magnetically-labeled PDGFRα^+^ cells were sorted by the autoMACS Pro Separator (Miltenyi Biotec) at 4°C.

### Flow cytometry

CMs were dissociated by incubating them in 0.25% Trypsin-EDTA (#25200, Thermo Fisher Scientific) for 10-15min at 37°C. To eliminate dead cells, cells were stained using the LIVE/DEAD fixable Aqua dead cell staining kit (#L34957, Thermo Fisher Scientific). CMs were fixed in 4% paraformaldehyde (PFA) for 10–15 mins and washed with PBS(-) twice. Then, cells were additionally permeabilized with 90% methanol for 15 min at room temperature and washed with PBS(-) twice. After that, cells were stained with an anti-cardiac isoform of Troponin T (cTnT) antibody (1:50, #MS-295-P, mouse monoclonal, clone 13–11, Thermo Fisher Scientific) labeled with Alexa-488 using the Zenon technology (#Z25002, Thermo Fisher Scientific) according to the manufacturer’s instructions, diluted in PBS(-) supplemented with 2% BSA and 0.2% Triton-X, for 30 min at room temperature. Cells were then washed twice with PBS(-) supplemented with 2% BSA and 0.2% Triton-X, and subjected to FACS analysis. For Ki67 staining, a phycoerythrin (PE)-conjugated mouse Anti-Ki67 antibody (1:50, #556027, BD Bioscience) was co-added with the Alexa-488-labeled anti-cTnT antibody. Mouse IgG1 (#557273, BD Bioscience) and PE-conjugated mouse IgG1 (#556027, BD Bioscience) were used as isotype controls. The staining was performed in PBS with 2% BSA and 0.2% Triton-X overnight at 4°C. Stained cells were analyzed using the Aria II flow cytometer (BD biosciences, Franklin Lakes, NJ). Data was collected from at least 10,000 events.

### Fluorescent microscopy

CMs were dissociated and plated onto chambered cell culture slides (0.86–1.43 × 10^5^ cells/cm^2^) (Falcon 8-well culture slide, #354118, corning) which were pre-coated with Poly-L-lysine and 0.5% gelatin (described in the section about the long-term culture of CMs), and were allowed to attach for 5–6 days. Cells were then fixed in 4% PFA for 15 mins at room temperature, blocked in PBS containing 2% BSA and 0.2% Triton-X for 2h at room temperature, and stained with anti-cTnT (1:200, rabbit polyclonal, Abcam, Cambridge, UK), anti-MLC-2a (1:400, #311011, mouse monoclonal, clone 56F5, Synaptic Systems, Germany), anti-MLC-2v (1:200, #10906-1-AP, rabbit polyclonal, ProteinTech), or PE-conjugated mouse anti-Ki67 (1:50, #556027, BD Bioscience) antibodies overnight at 4°C in PBS containing 2% BSA and 0.2% Triton-X. To establish the background fluorescence levels and set up the threshold for positive fluorescence, Mouse IgG2b (#14-4732-85, eBiosciences), and rabbit IgG (#02–6102, Life Technologies), or PE-conjugated Mouse IgG1 (#556027, BD Bioscience) were used as isotype controls. Cells were washed four times for 10 mins each, blocked with PBS containing 2% BSA and 0.2% Triton-X and then incubated overnight at 4°C in the dark with Alexa Fluor 488-conjugated goat anti-mouse IgG2b (1:500, #A-21141, Thermo Fisher Scientific), or Alexa Fluor 647-conjugated goat anti-rabbit IgG (H+L) (1:500, #A-21245, Thermo Fisher Scientific) secondary antibodies, and with DAPI (4, 6 diamidino -2-phenylindole; Thermo Fisher Scientific)). The stained samples were imaged using the Biorevo BZ-9000 All-In-One Fluorescence Microscope (Keyence, Osaka, Japan). Each cell area was manually segmented under visual inspection, and then the average expression of MLC-2a and MLC-2v in each single cell was calculated by dividing the total fluorescent intensity of a single cell by the area of the cell using the BZ-II Analyzer software BZ‐H1M (Keyence, Osaka, Japan). The fluorescent intensities of the isotype control staining were set as background. We evaluated a total of 348 cells on d21 and 308 cells on d91, and classified them into 3 cell types: those with stronger staining for MLC-2a than for MLC-2v (A^high+^ V^low+^), those with stronger staining for MLC-2v than for MLC-2a (A^low+^ V^high +^) and MLC-2a single positive cells (A+/V-).

For double staining of cTnT and α-actinin, CMs were dissociated and plated onto 96-well optical-bottom plates with a polymer base (#165305, Thermo Fisher Scientific) which were pre-coated with matrigel (#354230, Growth factor reduced, 1:60 dilution; Corning), at a density of 5.0 × 10^3^ cells per well (1.6 × 10^4^ cells/cm^2^), and were allowed to attach overnight. Cells were fixed in 4% PFA for 15 mins at room temperature, washed five times for 3 mins each, blocked using blocking buffer (Protein Block Serum-Free, #X0909, DAKO Agilent, CA, USA) for 15 mins at room temperature, and stained with anti-cTnT (1:400, rabbit polyclonal, Abcam, Cambridge, UK) and anti-α-actinin (1:500, #A7811, mouse monoclonal IgG1, clone EA-53, Sigma, Germany) antibodies in Antibody Diluent (#50809, DAKO Agilent) overnight at 4°C. Cells were washed five times for 3 mins each, and then incubated for 1 hour at room temperature in the dark with Alexa Fluor 546-conjugated goat anti-mouse IgG (H + L) (1:500, #A-11029, Thermo Fisher Scientific) and Alexa Fluor 488-conjugated goat anti-rabbit IgG (H+L) (1:400, #A-11035, Thermo Fisher Scientific) secondary antibodies. Cells were washed five times for 3 mins each and then stained with the Fluoro-KEEPER Antifade Reagent, Non-Hardening Type and DAPI (#12745–74, nacalai tesque, Kyoto, Japan). Stained images were photographed using a confocal microscope (Zeiss LMS 700, Carl Zeiss, Oberkochen, Germany).

### Quantitative Polymerase Chain Reaction (qPCR)

Total RNA was extracted from d21 and d91 CMs using the RNeasy Mini Kit (#74104, Quiagen, Hilden, Germany) according to the manufacturer’s instructions. RPS18 was used to normalize gene expressions. Quantitative PCR was performed using the Power SYBR Green PCR Master mix (#4367659, Thermo Fisher Scientific) on the StepOnePlus system (Thermo Fisher Scientific) and with the Delta Delta Ct method. Forward and reverse primer sequences are shown in S1 Table.

### Electrophysiology

We prepared CMs on d21 and d91. For single cell patch-clamp analysis, CMs were dissociated using 0.25% Trypsin in EDTA and re-seeded on fibronectin-coated coverslips in RPMI 1640 medium with B27 supplement and 10 μM Y27632. Patch clamp recordings were performed 4–6 days after plating. Coverslips were transferred to a patch clamp recording chamber, where spontaneous beating cells were subjected to whole-cell patch clamp at 35–37°C for this study. Electrophysiological measurements were carried out using the Axopatch200B amplifier and the Digidata 1440A interface (Molecular Devices, CA). The physiological bathing solution contained 2.0 mM CaCl_2_, 1.0 mM MgCl_2_・6H_2_O, 0.28 mM MgSO_4_, 5.0 mM KCl, 0.22 mM KH_2_PO_4_, 27.0 mM NaHCO_3_, 120 mM NaCl, 0.84 mM Na_2_HPO_4_, 5.55 mM D-Glucose; with pH adjusted to 7.4 by the addition of NaOH. Patch pipettes were pulled, fire-polished to final tip resistances of 2 to 4 mΩ, and then filled with a pipette solution containing 130 mM KOH, 130 mM L-Aspartic acid, 20 mM KCl, 1 mM MgCl_2_-6H_2_O, 5 mM NaCl, 10 mM EGTA, 5 mM Mg-ATP, 10 mM HEPES; pH was adjusted to 7.2 with KOH. Data was analyzed using the pClamp software (Molecular Devices). Action potential (AP) characterization was calculated from an average of 10 subsequent APs. The criteria to classify the APs into ventricular-like, atrial-like and nodal-like have been defined in a previous report [16, 30]. Non-ventricular CMs (APD30-40 / APD70-80 ratio: < 1.5) were subsequently classified into nodal-like CMs (dV/dt max < 10 mV/ms) and atrial-like CMs (dV/dt max ≥ 10 mV/ms). To further distinguish between early and late ventricular-like CMs (APD30-40 / APD70-80 ratio: ≥ 1.5), we set a broader value at 30 mV/ms, which was the mean + 2SD of dV/dt max for d21 CMs.

### Multi-electrode array experiment

For demonstrating the pharmacological utility of long term-cultured CMs, we recorded extracellular field potentials (FPs) in multielectrode arrays using the MED system (Alpha MED Scientific, Osaka, Japan) and evaluated cellular drug responses. The recording areas of multi-electrode probes (MED64 probe: MED-P515A; Alpha Med Scientific, Osaka, Japan) were coated with 2 μL of 50 μg/mL fibronectin (BD) and incubated at 4°C overnight. hiPSC-derived CMs were then dissociated using 0.25% Trypsin-EDTA for 10 mins at 37°C, re-plated onto the multi-electrode probes at a density of 3.0 × 10^4^ cells in 2 μL of αMEM+10% FBS containing 10 μM Y27632, and incubated for 3 to 5 h at 37°C under a 5% CO2 atmosphere. Each single-well MED64 probe was then filled with 1–2 mL of αMEM+10% FBS containing 10 μM Y27632, and cells cultured for 2–3 days. Medium was then completely replaced by RPMI1640 with B27 supplement and, after that, half of the medium was changed every 2 days. 3 days after the initial placement, spontaneous EFPs were recorded in each microelectrode.

We measured FPs according to a previous report, with some modifications [31]. In brief, samples were equilibrated for at least 30 min in a CO_2_ incubator in 2 mL of fresh medium prior to the measurements. After equilibration, the MED probes were maintained at 37°C with thermo-control systems and covered with a lid through which aeration (O_2_:CO_2_:N_2_ = 20%: 5%: 75%) was administered. FPs from spontaneously beating samples were filtered with a vessel 1–1000 Hz band-pass filter using the MED64 System. FP duration (FPD) was defined as the interval from the first peak (depolarization) to the second peak (repolarization). After recording the basal state, 2 μL of dimethyl sulfoxide (DMSO; Wako) were added and FPs were recorded for 10 min. Then, E-4031 (Wako) or Chromanol 293B (Sigma-Aldrich) were cumulatively added to obtain the target concentrations, and FPs were recorded in the same manner as for DMSO administration. For E-4031, four drug concentrations (0.3 nM, 3 nM, 10 nM, 30 nM) were selected to evaluate dose-dependent effects, while for Chromanol 293B, three drug concentrations (3 μM, 10 μM, 30 μM) were selected. At each concentration, the FP was recorded for 10 min, and the last 30 beats recorded by the electrode with the most evident and easy-to-analyze peak among all 64 electrodes were extracted as a dataset for FPD and waveform analysis, according to a previous report [32]. The FPD values from the last 30 beats at each drug concentration were averaged.

### Transmission Electron Microscopy (TEM)

hiPS-derived CMs were examined using electron microscopy with support from the Center for Anatomical, Pathological and Forensic Medical Research, Kyoto University Graduate School of Medicine, and a contract analysis with Hanaichi Ultrastructure Research Institute, Co., Ltd. (HURI), Japan. At Kyoto University, cells were fixed in 4% glutaraldehyde in PBS overnight at 4°C and washed 3 times with PBS. Post-fixation was done using OsO4 (1% for 1 min, and 0.5% for 20 min at 4°C) in PBS, dehydrated in ethanol and propylene oxide, and embedded in Luveak 812 (Nacalai Tesque, Japan). Ultrathin sections were then cut using an ultramicrotome (Leica, Heidelberg, Germany) and observed with TEM (H-7650; Hitachi, Japan). At HURI, cells were fixed in 2% glutaraldehyde in 0.1 M phosphate buffer at pH7.4, at 4°C, overnight, and then washed 3 times with 0.1 M phosphate buffer at pH7.4. Post-fixation was done in 2% Osmium tetra-oxide/distilled water for 2 hours at 4°C, dehydrated in ethanol and propylene oxide, and embedded in EPON812 (TAAB Laboratories Equipment, UK). Ultrathin sections were cut using an ultramicrotome (REICHERT-NISSEI, ULTRA CUTS, Germany) and observed with TEM (H-7600; Hitachi). Sarcomere length (distance between Z-bands) were measured manually in randomly selected electron microscopy images.

### Statistical analysis

The data was processed using Graph Pad Prism software (Ver5.0, GraphPad Software, San Diego, CA). Comparisons between two and three groups were made with Mann-Whitney tests or with one-way analysis of variance (ANOVA) followed by Tukey-Kramer test as post hoc, respectively. Values are shown as means ± SD. P values < 0.05 were considered significant.

## Results

### Induction and long-term maintenance of high-purity hiPSC-derived CMs

Based on our previous 2-dimensional culture method for CM differentiation from hPSCs, we first induced mesoderm populations with Act-A, BMP4, and bFGF. We chose PDGFRα^+^ cells at differentiation day 5 (d5) as a cell population that is responsive to CM differentiation cues. PDGFRα^+^ cells were purified by MACS resulting in more than 95% purity, and then re-cultured with Wnt inhibitors to potently drive PDGFRα^+^ cells towards the CM lineage (Fig 1).

**Fig 2:**
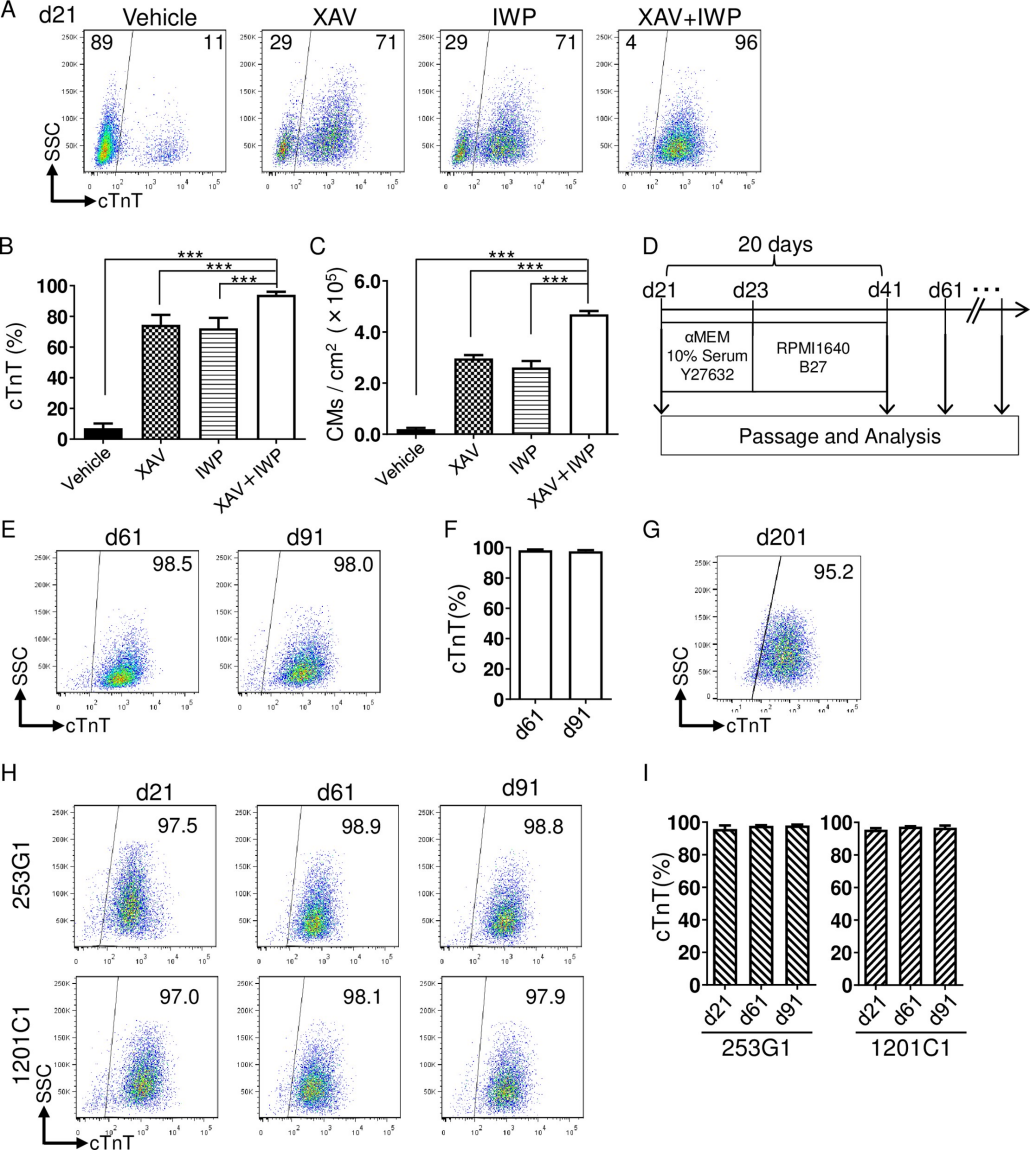
Induction and maintenance of hiPSC-derived CMs with high-purity. (A) Representative flow cytometry analysis of cTnT (and side scatter plot (SSC)) on d21 induced by combinations of Wnt signal inhibitors (XAV939 and IWP4). The percentages of cTnT^+^ and cTnT^-^ cells are indicated. (B) Quantitative analysis of cTnT^+^ CMs on d21. Data are means ± SD. ***p < 0.001 by one-way ANOVA followed by Tukey-Kramer post hoc test (n = 4). (C) CM yield on d21. Data are means ± SD. ***p < 0.001 by one-way ANOVA followed by Tukey-Kramer post hoc test (n = 4). (D) Scheme of CM long-term maintenance protocol. CMs were re-cultured under αMEM medium with 10% FBS and Y27632, for the initial 2 days, followed by a serum-free condition (RPMI1640+B27) for 18 days. CMs underwent passage every 20 days. (E) Representative flow cytometry analysis of cTnT in 836 B3 cell line on d61, and d91. Percentages of cTnT^+^ cells are indicated. (F) Quantitative analysis of cTnT^+^ CMs in 836 B3 cell line on d61, and d91. (n = 4) (G) Representative flow cytometry analysis of cTnT in 836 B3 cell line on d201. Percentages of cTnT^+^ cells are indicated. (H) Representative flow cytometry analysis of cTnT in 253G1 and 1201C1 cell lines on d21, d61, and d91. Percentages of cTnT^+^ cells are indicated. (I) Quantitative analysis of cTnT^+^ CMs in 253G1 and 1201C1 cell lines on d21, d61, and d91. (253G1; n = 4, 1201C1; n = 5).

In this protocol, we selected two small molecule Wnt inhibitors; XAV939 and IWP4, which have different mechanisms of action. XAV939 is a tankyrase inhibitor that stabilizes GSK3β, resulting in Wnt inhibition [33], while IWP4 inhibits porcupine, which in turn reduces Wnt ligand secretion and ligand-receptor binding affinity [34]. Simultaneous addition of XAV939 and IWP4 robustly induced cTnT^+^ CMs from PDGFRα^+^ cells on d21 with approximately 95% efficiency (cTnT^+^ percentages: Control (without Wnt inhibitors); 7.1 ± 3.1%, XAV; 74.5 ± 6.5%, IWP; 72.2 ± 6.8%, XAV+IWP; 94.1 ± 2.0%) (Fig 2A and 2B). This dual Wnt inhibition led to an approximately 25 times higher yield in CM numbers, compared to the control (Control; 0.2 ± 0.1 × 10^5^ cells/cm^2^, XAV; 3.0 ± 0.3 × 10^5^ cells/cm^2^, IWP; 2.6 ± 0.5 × 10^5^ cells/cm^2^, XAV+IWP; 4.7 ± 0.3 × 10^5^ cells/cm^2^) (Fig 2C). To confirm whether this high CM purity can be continuously maintained during long-term culture, we re-plated CMs on d21 in αMEM medium with FBS and ROCK inhibitor (Y27632) for only the first two days to ensure CM attachment to the dish, followed by a switch to RPMI1640 medium with B27 supplement (RPMI1640+B27) for 18 days, and repeated this cycle every 20 days (Fig 2D). The percentage of cTnT^+^ CMs was assessed by flow cytometry. The high-purity of the CM population on d21 (>95–98%) was maintained in this simple culture condition for approximately 2–3 months (Fig 2E and 2F). Notably, a high-purity CM population (> 95%) was still maintained even at d201 without any CM purification step (Fig 2G). We verified our protocol with two other cell lines, 253G1 and 1201C1 [28, 29]. We also observed CM induction with more than 95% efficiency in these two lines and maintained that purity until d91 (Fig 2H and 2I). These data indicate that our protocol can robustly achieve CM differentiation with high efficiency as well as long-term maintenance of high-purity CMs in various hiPSC lines, regardless of their origin and derivation.

Undifferentiated hPSCs were plated 2 or 3 days before the induction of differentiation (-d2~3) on Matrigel-coated dishes in mouse embryonic fibroblast conditioned medium (MEF-CM). A matrigel overlayer was applied on -d1. After the induction of PDGFRα^+^ cells with Activin-A followed by BMP4 and bFGF in RPMI1640 medium including B27 supplement without insulin (B27 minus insulin), PDGFRα^+^ cells were purified using MACS and re-cultured on gelatin or matrigel-coated plates in FBS-containing medium with Wnt signal inhibitors (XAV939: 10 μM and IWP4: 5 μM) and a ROCK inhibitor (Y-27632) for 2 days. After that, medium was changed to a serum free condition (RPMI1640+B27) with a minimal dose of Wnt signal inhibitors (XAV939: 0.25 μM and IWP4: 0.125 μM). Following this protocol, highly pure CMs were induced and maintained in the long-term.

### Proliferation capacity of hiPSC-derived CMs

**Fig 3:**
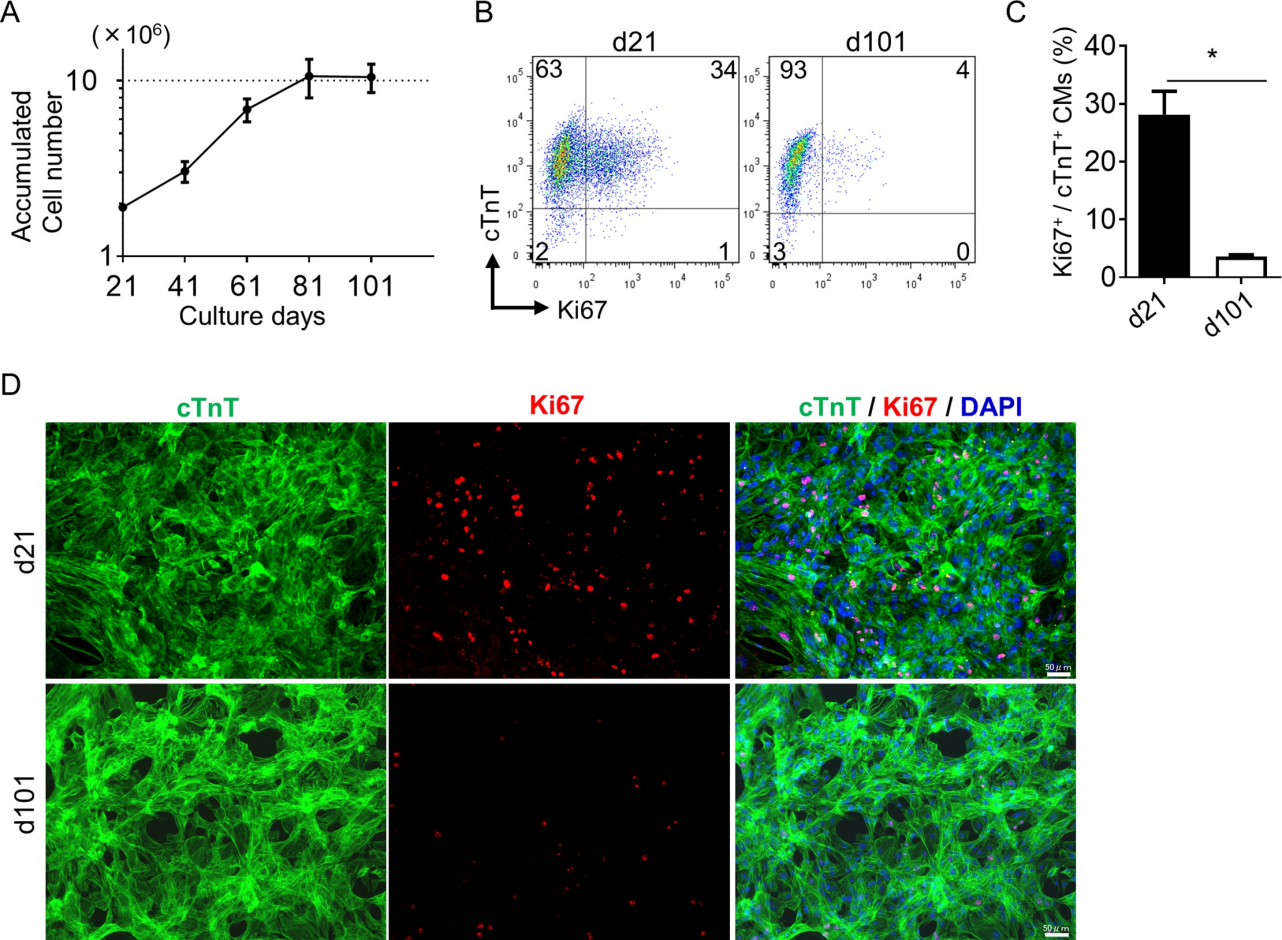
Proliferation capacity of hiPSC-derived CMs. (A) Growth profile of CMs from d21 to d101. CMs on d21 were seeded at 2.0 × 10^5^ cells/cm^2^ (1.9 × 10^6^ cells per well of a 6-well plate). Cells were passaged every 20 days and plated at the same cell density. The accumulated cell number was calculated from cell counts during subsequent passages. Data are means ± SD. (n = 4). (B) Representative flow cytometry analysis of cTnT and Ki67 on d21 and d101. Numbers in each quadrant represent the respective percentages of cells. (C) Quantitative analysis of Ki67^+^/cTnT^+^ CMs. Data are means ± SD. *p < 0.05 by Mann-Whitney test (n = 4). (D) Representative immunostaining of d21 and d100 CMs for cTnT (Green), Ki67 (Red), and DAPI (Blue). Scale bars, 50 μm.

To assess the proliferation capacity of induced human CMs, we continuously cultured CMs and counted their numbers from d21 to d101 with sequential passages every 20 days. A constant increase in the number of CMs was clearly observed until d81. A single CM on d21 was capable of generating approximately 5.6 ± 1.4 CMs following 60 days of culture (Fig 3A). We quantitatively examined the proliferation capacity of these CMs by Ki67 staining and flow cytometry (Fig 3B). Although Ki67^+^ proliferating CMs were approximately 30% on d21 (d21; 28.1 ± 4.0%), they declined to around 4% or less by d101 (d101; 3.6 ± 0.7%) (Fig 3C). Similar results were confirmed by immunostaining of Ki67 and cTnT (Fig 3D). The proliferative capacity of hiPSC-derived CMs was maintained for approximately 2 months after the appearance of CMs, and disappeared between d81 and d101 under this culture condition.

### Chamber specification and structural maturation of hiPSC-derived CMs after long-term culture

**Fig 4:**
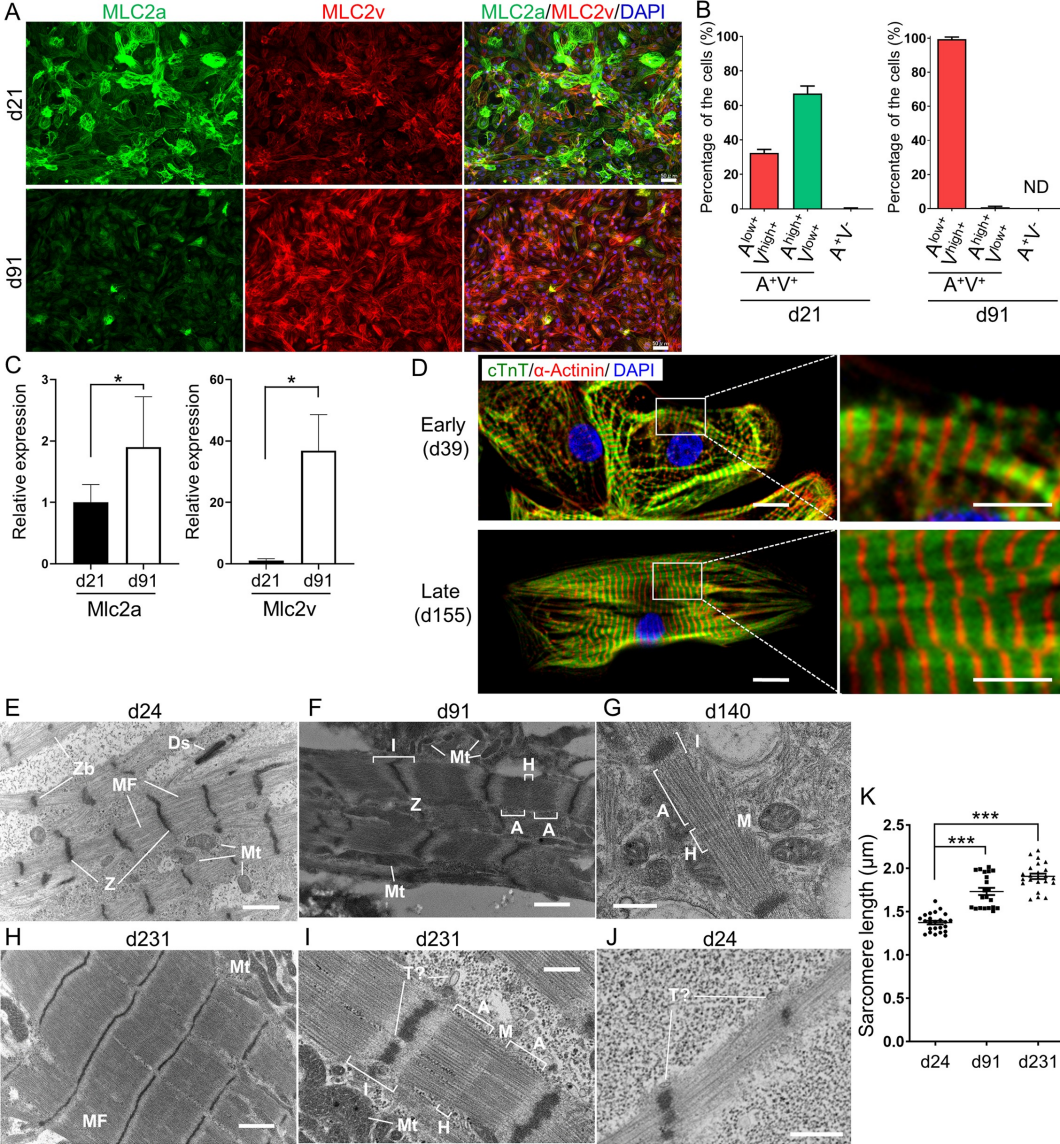
Structural maturation and chamber specification of hiPSC-derived CMs after long term culture. (A, B) Immunostaining of MLC-2a and MLC-2v. (A) Immunofluorescent staining of MLC-2a (Green), MLC-2v (Red), and DAPI (Blue) in d21 and d91 CMs. Scale bar, 50 μm. (B) Quantification of MLC2a and MLC2v immunostaining of d21 and d91 CMs. Data are means ± SD. (n = 4). A+V+: MLC-2a-positive MLC-2v-positive, ventricular CMs; A^high+^ V^low+^: MLC-2a-high positive MLC-2v-low positive, immature ventricular CMs; A^low+^ V^high +^: MLC-2a-low positive MLC-2v-high positive, relatively mature ventricular CMs; A+ V-: MLC-2a-positive MLC-2v-negative, atrial CMs; ND: not detected. (C) qPCR of relative mRNA expressions of Mlc2a and Mlc2v in CMs on d21 and d91. The mean of d21 was set as 1. **p<0.01, *p<0.05, n = 6 with technical triplicates for each. (D) Double immunofluorescent staining of cTnT (green) and α-actinin (red) on d39 and d155. Right panels: higher magnification views of boxed areas in the left panels. Scale bars in the left panels: 10 μm. Scale bars in the right panels: 5 μm. (E-J) Transmission electron microscopy images of CMs. (E) CMs on d24. CMs contained myofibrils (MF) that lacked clear alignment or an organized sarcomeric pattern. Z-bodies (Zb), CMs connected by desmosomes (Ds) and mitochondria (Mt) can be observed. (F) CMs on d91. Sarcomeres show clearly aligned Z-disks (Z) and organized A- and I-bands with a clear H-zone (H). Mitochondria (Mt) are located adjacent to sarcomere structures. (G) CMs on d140. Faint M-lines starting to form. (H) CMs on d231. Clear muscle-like alignment of myofibers and mitochondria can be observed. (I) CMs at d231. Clear sarcomeric structures with Z-, H-, I-, A-, and M-bands and well established mitochondria can be observed. T?: T-tubule-like structure. (J) CMs on d24. T-tubule-like structures along Z-bands (T?). Scale bars: 1μm (E, F, H), 400 nm (G, I), 600 nm (J). (K) Sarcomere length in CMs on d24, d91, and d231. ***p<0.001, n = 24 (d24), 21 (d91), 23 (d231), respectively.

Next, we evaluated the structural maturation and specification of CMs. We examined the atrial and ventricular specification of hiPSC-derived CMs. In human embryonic and adult hearts, myosin light chain-2a (MLC-2a) is expressed in both the atrium and the ventricle, whereas myosin light chain-2v (MLC-2v) is selectively expressed in the ventricle [35, 36]. The expression of MLC-2a in immature MLC-2v^+^ ventricular CMs gradually decreases as CMs mature [37]. In our experiments, MLC-2v expression was heterogeneous but positive in almost all CMs on d21 and d91 (Fig 4A and 4B). On d21, CMs with higher expression of MLC-2a than MLC-2v (A^high+^ V^low+^) were observed, but that higher MLC-2a expression had almost completely disappeared by d91 (d21; 67.0 ± 4.3%, d91; 0.6 ± 1.3%). On the contrary, cells expressing higher MLC-2v levels (A^low+^ V^high +^) increased in number and dominated the population by d91, compared to d21 (d21; 32.5 ± 4.0%, d91; 99.4 ± 1.3%). MLC-2a^+^ MLC-2v^-^ (A^+^V^-^) atrial CMs were very rare (d21; 0.3 ± 0.6%, d91 A^+^V^-^; not detected). We further confirmed this ventricular specification at the mRNA expression level. Although Mlc2a mRNA expression was largely comparable between d21 and d91 CMs, Mlc2v expression was drastically increased in d91 CMs to approximately 37 times that in d21 CMs (Fig 4C). These results suggest that our differentiation protocol strongly and specifically induced ventricular-type CMs.

Next, we examined the structural maturation of CMs. Double immunofluorescent staining of cTnT and α-actinin clearly showed a tendency to morphological maturation (Fig 4D). Though sarcomere structures were formed in both d39 and d155 CMs, myofibers on d39 were not clearly aligned and cells showed a polygonal appearance, suggesting that CMs were still immature. On the other hand, some CMs on d155 exhibited a rod shape with clear myofiber alignment resembling mature ventricular CMs. Transmission electron microscopy (TEM) analysis also demonstrated the gradual maturation of CMs at the ultrastructural level. CMs on d21 contained myofibrils (MF) that lacked a clear alignment or an organized sarcomeric structure with scattered Z-body (Zb) patterns. Mitochondria exhibited round immature shapes (Fig 4E). On the other hand, CMs on d91 showed a more mature ultrastructure (Fig 4F). Sarcomeres showed clearly aligned Z-disks and organized A- and I-bands with H-zones. CMs on d140 featured faint M-lines starting to form (Fig 4G). Mitochondria showed a tendency to increase in number and grow in size with a complex inner structure and location alongside sarcomeres. On d231, massive well aligned myofibrils with clear sarcomere formation and mitochondria alongside sarcomeres were observed (Fig 4H). In addition to a clear sarcomeric structure with Z-, I-, A-, H-, and M-bands and well established mitochondria with mature inner composition, transverse tubule (T-tubule)-like structures were found along the Z-bands, where T-tubules are located in CMs (Fig 4I). Measurements from electron microscopy images demonstrated that sarcomere length was increased during long-term culture from 1.37 ± 0.10 μm on d24 to 1.73 ± 0.19 μm on d91 and to 1.91 ± 0.15 μm on d231 (Fig 4K). These results indicate that the structural maturation of CMs gradually progresses during long-term culture.

### Electrophysiological characterization and maturation of CMs after long-term culture

**Fig 5:**
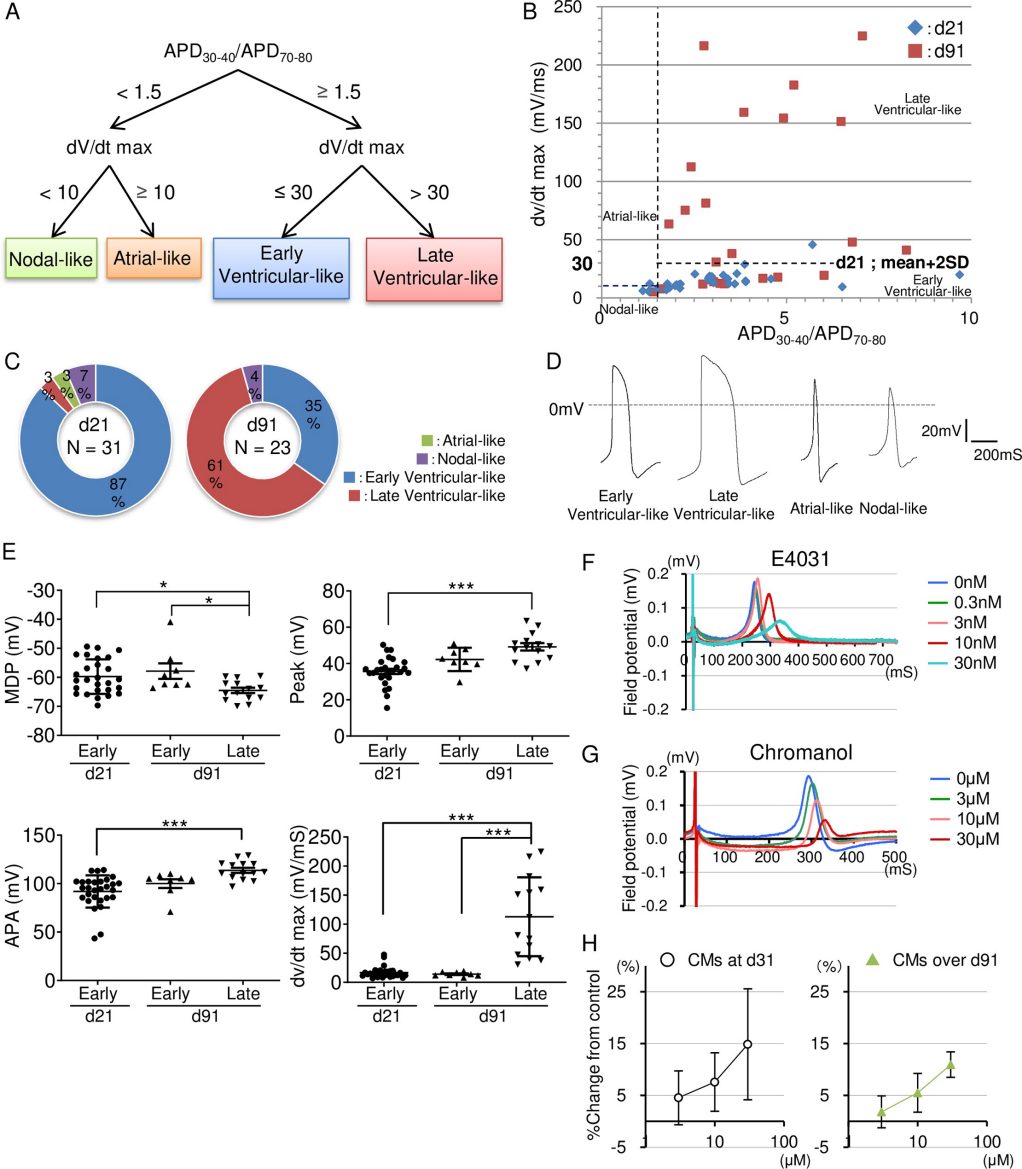
Electrophysiological characterization and maturation of CMs after long-term culture. (A) Criteria for CM subtype classification. (B) Relationship between APD30-40/APD70-80 ratio and dV/dt max on d21 and d91 CMs. (C) Pie charts illustrating the distribution of early ventricular-, late ventricular-, atrial- and nodal-like APs on d21 (n = 31 from three independent experiment), and d91 (n = 23 from three independent experiment). (D) Representative action potentials of early and late ventricular-like and nodal-like CMs. (E) Comparison of action potential parameters between early ventricular-like and late ventricular-like CMs on d21(n = 28 from three independent experiments) and d91 (n = 22 from three independent experiments). (n = 3). *p < 0.05, ***p < 0.001 by one-way ANOVA followed by Tukey-Kramer post hoc test. (F) Administration of E4031 (Ikr blocker) prolonged FPD in a dose-dependent manner at 0–30 nM in CMs. (G) Administration of Chromanol (Iks blocker) prolonged FPD in a dose-dependent manner at 0–30 μM in CMs. (H) Homogeneous response to Chromanol in a time-dependent manner. d31 (n = 16), over d91 (n = 8).

To examine the degree of electrophysiological maturation of CMs on d21 and d91, we examined action potentials (APs) using patch clamp analysis. APs were recorded from spontaneously beating single CMs on d21 and d91 (S2 and S3 Tables). We first categorized ventricular-like CMs and non-ventricular CMs according to their AP duration (APD) 30-40/APD70-80 ratio (≥ 1.5) as previously reported [30]. Then, according to the maximal rate of depolarization (dV/dt max), we further classified non-ventricular CMs into nodal-like and atrial-like, and ventricular-like CMs into early and late ventricular-like cells (Fig 5A). Briefly, non-ventricular CMs (APD30-40 / APD70-80 ratio: < 1.5) were classified into nodal-like CMs (dV/dt max < 10 mV/ms) and atrial-like CMs (dV/dt max ≥ 10 mV/ms) [38, 39]. To distinguish the early and late ventricular-like CMs, we set a broader value at 30 mV/ms, which was the mean plus two standard deviations of dV/dt max in CMs on d21 (Fig 5B). Ventricular-like CMs were further classified into early ventricular-like CMs (dV/dt max < 30 mV/ms) and late ventricular-like CMs (dV/dt max ≥ 30 mV/ms). The overall distribution of CMs on d21 and d91 is displayed in Fig 5B. Consistent with the results of MLC-2a and MLC-2v immunostaining (Fig 4A and 4B), the majority of CMs were categorized as ventricular-like CMs (90% at d21, 96% at d91) (Fig 5C and 5D). Late ventricular-like CMs increased from 3% (d21) to 61% (d91), indicating that relative electrophysiological maturation as well as structural maturation occurred during long-term culture. Only one cell among the total 54 cells analyzed (on d21 and d91) was categorized as an atrial-like CM and few cells showed nodal-like CM features (7% and 4% on d21 and d91, respectively) (Fig 5B–5D). In addition to the dV/dt max characterization, late ventricular-like CMs on d91 had deeper depolarized maximum diastolic potentials (MDPs), and larger peak voltages and action potential amplitudes (APAs) compared to CMs on d21, indicating that ventricular-like CMs were predominantly induced and also underwent gradual electrophysiological maturation during long-term culture (Fig 5E, S2 and S3 Tables).

Finally, to demonstrate the utility of long term-cultured CMs for pharmacological studies, we examined the effects of selective ion channel inhibitors, the human Ether-a-go-go Related Gene (hERG) channel blocker, E4031, and the KCQN1 channel blocker, Chromanol-293B (Chromanol). We treated CMs with E4031 or Chromanol and examined their electrophysiological features by field potential duration (FPD) recorded using multi-electrode arrays (MEAs) [17]. E4031 and Chromanol have been reported to induce QT prolongation on the electrocardiogram by inhibiting the delayed-rectifying K^+^ current (*I*_*Kr*_) and the slow delayed rectifier current (*I*_*Ks*_), respectively. Apparent dose-dependent FPD prolongations by E4031 and Chromanol, which correspond to QT prolongations on the electrocardiogram, were observed in CMs (Fig 5F and 5G). *I*_*kr*_ has been detected in hESC-derived immature CMs whereas *I*_*Ks*_ has been reported to show low expression levels in immature CMs and increased levels in mature CMs [30, 40, 41]. We compared the difference in the response to Chromanol among CMs on d31 and over d91 (Fig 5H). Although the average % change of FPD values in each dose was not apparently different, the deviation of these values tended to be smaller in late ventricular-like CMs over d91 (S4 Table). These results suggest that during maturation in long-term culture, CMs became more stable and homogenous in their electrophysiological features, implying potential utility for pharmacological studies.

## Discussion

In the present study, we established an efficient method to obtain hiPSC-derived high-purity CM populations without the need for CM purification steps. Moreover, these populations maintain their high-purity (>95%) for over 200 days of culture (long-term culture). The majority of CMs were induced specifically into a ventricular phenotype and exhibited gradual structural and electrophysiological maturation. These high-purity CM populations with a relatively homogenous and matured ventricular phenotype could potentially have an advantage over immature CMs for pharmacological studies.

We previously reported the induction of pure ECs based on the SE method [18]. Adapting this approach to CM differentiation, we collected responder cells by purifying PDGFRα^+^ cells on d5, and strongly committed these cells to the CM lineage. We did so by inhibiting canonical Wnt signaling with two Wnt inhibitors, XAV939 and IWP4 [17]. This CM induction approach, based on the SE method, yielded high-purity hiPSC-derived CM populations that can maintain their purity during long term maintenance without the need for CM purification steps (Fig 1). The removal of aggressive purification procedures prevents potential CM damage that could affect their purity and viability. In addition, our method allows for the long-term maintenance and expansion of pure CM populations across several hPSC lines with no gene modifications, such as the introduction of antibiotic-resistant genes, required. Since our differentiation method yields pure and viable high-quality CMs and is expandable to a broad range of hPSC lines (including disease lines), it could be very valuable for pharmacological testing. Furthermore, the elimination of cells that are not susceptible to target differentiation stimuli during the earlier differentiation stages successfully induced highly pure ECs [18] and CMs (this study), suggesting that the SE method approach could be useful to generate other hPSC-derived cell populations with high purity and long-term maintenance capacity.

In our experiments, the proliferative capacity of CMs was gradually lost as CMs matured (Fig 3). Similarly, in human heart development, CMs almost completely lose their proliferative capacity after the first few postnatal months [42]. Although the relationship between the proliferative capacity of human embryonic and postnatal CMs and hiPSC-derived CMs remains uncertain, this decreased proliferative capacity of hiPSC-derived CMs throughout maturation may recapitulate postnatal heart development [43]. Another study reported that hiPSC-derived CM proliferation in culture and after transplantation into mouse hearts seemed to cease at around 3 months [44], supporting our results. The proliferative capacity of hiPSC-derived CMs seems to be restricted within this period.

There was one report about hiPSC-derived CMs structurally evaluated by TEM along 1 year of *in vitro* culture [37]. TEM demonstrated gradual sarcomeric formation with Z-disks and I-bands by 180 days and with H-zones, and M-lines by 1 year. However, T-tubules, which are indispensable in adult CMs for rapid excitation-contraction coupling, were never observed. In our study, T-tubule-like structures were observed on d231 (Fig 4I). We consider our highly pure ventricular CM culture may contribute to T-tubule formation. Recently, engineered cardiac tissues made from hiPSC-derived CMs and fibroblasts in fibrin hydrogels and subjected to stretch and auxotonic contractions for just four weeks showed structural maturation, including T-tubule formation [45]. In our experiments, we observed immature T-tubule-like structures along with Z-bands as early as d24 (Fig 4J). Based on this evidence, we consider that T-tubule formation may be initiated at an earlier stage of CM differentiation than was previously thought. Currently, the mechanisms involved in the generation and maturation of T-tubules in human CMs are largely unknown. Our method may provide a useful platform to manipulate and study CM maturation, including T-tubule formation.

As for the electrophysiological maturation of high-purity long-term cultured CMs, there was a prominent change in dV/dt max between CMs on d21 and d91 (Fig 5B). Although there was some heterogeneity among samples, some CMs on d91 showed more than 200 mV/ms. These values are still lower than those previously reported for human adult CMs (around 300–400 mV/ms) [46, 47], but are comparable to those of human PSC-derived CMs exhibiting signs of *in vitro* maturation (>150 mV/ms) [48, 49]. This tendency to electrophysiological maturation during long-term culture could be reflected in the formation of T-tubule-like structures like the ones observed in our electron microscopy analysis (Fig 4I). An increased expression and function of the I_*Ks*_ channel is also an indicator of CM maturation [30, 40, 41]. The response of late ventricular-like CMs to Chromanol was relatively stable and reproducible, suggesting that *I*_*Ks*_ functionally worked in CMs on d91. Additionally, the smaller standard deviations observed in d91 CMs suggest a less heterogeneous phenotype within the population in terms of electrophysiological maturation (Fig 5H).

Our differentiation protocol enabled us to obtain highly pure ventricular-like CMs with no specific selection steps. In previous reports, atrial specification of CMs derived from hESCs or hiPSCs has been mediated by retinoic acid or retinal aldehyde dehydrogenase 2 (which converts retinal aldehyde to retinoic acid) [50–52]. These *in vitro* studies were based on the mammalian heart development *in vivo* [53, 54]. As to the specification of the ventricular lineage, a higher ratio of ActA to BMP4 signaling (5 ng/mL of BMP and 12 ng/mL of ActA) during the initial stage of hPSC differentiation was required to induce a CD235a^+^ mesoderm population, which is considered a prerequisite for the generation of populations highly enriched in ventricular cardiomyocytes [50]. However, the percentage of CD235a^+^ cells is not necessarily predictive of the subsequent percentage of ventricular cardiomyocytes. Some ventricular cardiomyocytes were also induced from an isolated CD235a^-^ mesoderm population [50]. Some of our preliminary data showed that CD235a^+^ cells were just a small subset of our responder population (PDGFRα^+^ cells) (data not shown). In spite of this, ventricular-like cardiomyocytes were very specifically induced in our method, suggesting that the majority of CMs obtained in our method are in fact derived from a CD235a^-^ population. These contradictory conclusions may be due to the differences between these differentiation methods. For example, the initial differentiation condition in our protocol (100 ng/mL of ActA alone), is much different from that in the previous report. The methods for CM induction from the mesoderm stage onward are also largely different. Our method features a 2-D culture treated with Wnt inhibitors, being different from the cell aggregates with a Wnt inhibitor and VEGF utilized in [50]. The mechanisms and trajectory of the ventricular-specific induction achieved in our method are currently unclear.

In conclusion, we established a method for CM-specific induction and maintenance without CM purification steps which yielded high-purity ventricular CMs with a gradual tendency to structural and electrophysiological maturation. This method could offer a source of CMs for a number of applications, including cardiac toxicology testing and disease modeling as well as regenerative medicine.

## Supporting information

S1 Table List of forward and reverse primer sequences for qPCR. (DOCX)

S2 Table Summary of action potentials of cardiomyocytes on d21. (DOCX)

S3 Table Summary of action potentials of cardiomyocytes on d91. (DOCX)

S4 Table Summary of % change of FPDcF value. (DOCX)
