# Supplementary material for: Specific induction and long-term maintenance of high purity ventricular cardiomyocytes from human induced pluripotent stem cells
Source: PLoS One. 2020 Nov 2;15(11):e0241287. doi: 10.1371/journal.pone.0241287 (PMC7605685; doi:10.1371/journal.pone.0241287)
Supplement: S1 Table — (DOCX) [file pone.0241287.s001.docx]

**S1 Table. List of forward and reverse primer sequences for qPCR**

| Gene sequence | Sense | Antisense |
| --- | --- | --- |
| RPS18 | CCTTTGCCATCACTGCCATT | TGATCACACGTTCCACCTCA |
| Mlc 2a | GTCTTCCTCACGCTCTTTGG | CCACCTCAGCTGGAGAGAAC |
| Mlc 2v | ACAGGGATGGCTTCATTGAC | ATGCGTTGAGAATGGTTTCC |
