# Supplementary material for: Specific induction and long-term maintenance of high purity ventricular cardiomyocytes from human induced pluripotent stem cells
Source: PLoS One. 2020 Nov 2;15(11):e0241287. doi: 10.1371/journal.pone.0241287 (PMC7605685; doi:10.1371/journal.pone.0241287)
Supplement: S2 Table — (DOCX) [file pone.0241287.s002.docx]

**S2 Table. Summary of action potentials of cardiomyocytes on d21**

| **d21 (n = 31)** | | **MDP** | **Peak** | **APA** | **dV/dt Max** | **APD30-40 /APD70-80** |
| --- | --- | --- | --- | --- | --- | --- |
| **Ventricular-like** | **Early**  **(n = 27)** | -59.8±5.9 | 35.6±7.7 | 95.4±10.8 | 14.6±5.0 | 3.2±1.7 |
|  | **Late**  **(n = 1)** | -65.8 | 49.0 | 114.8 | 45.8 | 5.7 |
| **Nodal-like**  **(n = 2)** | | -52.4±0.7 | 19.3±5.6 | 71.7±4.9 | 6.0±0.3 | 1.2±0.1 |
| **Atrial-like**  **(n = 1)** | | -65.4 | 22.9 | 88.3 | 12.3 | 1.3 |

MDP: Maximum diastolic potential, Peak: Peak voltage, APA: Action potential amplitude, dV/dt max: maximal rate of depolarization, APD: AP duration at different levels of repolarization
