# Supplementary material for: Specific induction and long-term maintenance of high purity ventricular cardiomyocytes from human induced pluripotent stem cells
Source: PLoS One. 2020 Nov 2;15(11):e0241287. doi: 10.1371/journal.pone.0241287 (PMC7605685; doi:10.1371/journal.pone.0241287)
Supplement: S3 Table — (DOCX) [file pone.0241287.s003.docx]

**S3 Table. Summary of action potentials of cardiomyocytes on d91**

| **d91 (n = 23)** | | **MDP** | **Peak** | **APA** | **dV/dt Max** | **APD30-40 /APD70-80** |
| --- | --- | --- | --- | --- | --- | --- |
| **Ventricular-like** | **Early**  **(n = 8)** | -57.8±7.6 | 42.2±6.4 | 100.1±12.9 | 14.0±3.8 | 3.6±1.4 |
|  | **Late**  **(n = 14)** | -64.5±3.5 | 49.2±7.7 | 113.7±9.5 | 112.8±68.0 | 4.4±2.1 |
| **Nodal-like**  **(n = 1)** | | -49.5 | 26.7 | 76.2 | 5.1 | 1.4 |
