# Supplementary material for: Specific induction and long-term maintenance of high purity ventricular cardiomyocytes from human induced pluripotent stem cells
Source: PLoS One. 2020 Nov 2;15(11):e0241287. doi: 10.1371/journal.pone.0241287 (PMC7605685; doi:10.1371/journal.pone.0241287)
Supplement: S4 Table — (DOCX) [file pone.0241287.s004.docx]

**S4 Table. Summary of %Change of FPDcF value**

|  | **Chromanol concentration (μM)** | | | |
| --- | --- | --- | --- | --- |
|  | 0 | 3 | 10 | 30 |
| **CMs at d31 (n=16)** | 0 | 4.5±5.2 | 7.6±5.7 | 14.9±10.7 |
| **CMs over d91 (n=8)** | 0 | 1.8±3.1 | 5.5±3.7 | 10.9±2.5 |
